# The NanoFlow Repository

**DOI:** 10.1093/bioinformatics/btad368

**Published:** 2023-06-07

**Authors:** Jessie E Arce, Joshua A Welsh, Sean Cook, John Tigges, Ionita Ghiran, Jennifer C Jones, Andrew Jackson, Matthew Roth, Aleksandar Milosavljevic

**Affiliations:** Department of Molecular and Human Genetics, Baylor College of Medicine, Houston, TX 77030, United States; Translational Nanobiology Section, Laboratory of Pathology, Center for Cancer Research, National Cancer Institute, National Institutes of Health, Bethesda, MD 20892, United States; Translational Nanobiology Section, Laboratory of Pathology, Center for Cancer Research, National Cancer Institute, National Institutes of Health, Bethesda, MD 20892, United States; Department of Medicine, Beth Israel Deaconess Medical Center, Harvard Medical School, Boston, MA 02215, United States; Department of Medicine, Beth Israel Deaconess Medical Center, Harvard Medical School, Boston, MA 02215, United States; Translational Nanobiology Section, Laboratory of Pathology, Center for Cancer Research, National Cancer Institute, National Institutes of Health, Bethesda, MD 20892, United States; Department of Molecular and Human Genetics, Baylor College of Medicine, Houston, TX 77030, United States; Department of Molecular and Human Genetics, Baylor College of Medicine, Houston, TX 77030, United States; Department of Molecular and Human Genetics, Baylor College of Medicine, Houston, TX 77030, United States

## Abstract

**Motivation:**

Extracellular particles (EPs) are the focus of a rapidly growing area of exploration due to the widespread interest in understanding their roles in health and disease. However, despite the general need for EP data sharing and established community standards for data reporting, no standard repository for EP flow cytometry data captures rigor and minimum reporting standards such as those defined by MIFlowCyt-EV (https://doi.org/10.1080/20013078.2020.1713526). We sought to address this unmet need by developing the NanoFlow Repository.

**Results:**

We have developed The NanoFlow Repository to provide the first implementation of the MIFlowCyt-EV framework.

**Availability and implementation:**

The NanoFlow Repository is freely available and accessible online at https://genboree.org/nano-ui/. Public datasets can be explored and downloaded at https://genboree.org/nano-ui/ld/datasets. The NanoFlow Repository’s backend is built using the Genboree software stack that powers the ClinGen Resource, specifically the Linked Data Hub (LDH), a REST API framework written in Node.js, developed initially to aggregate data within ClinGen (https://ldh.clinicalgenome.org/ldh/ui/about). NanoFlow’s LDH (NanoAPI) is available at https://genboree.org/nano-api/srvc. NanoAPI is supported by a Node.js Genboree authentication and authorization service (GbAuth), a graph database called ArangoDB, and an Apache Pulsar message queue (NanoMQ) to manage data inflows into NanoAPI. The website for NanoFlow Repository is built with Vue.js and Node.js (NanoUI) and supports all major browsers.

## 1 Introduction

The role of extracellular particles (EPs) in many biological processes is coming to light through various publications and ongoing studies, fueling the growing interest in understanding their role in human health and disease. One of the significant challenges in studying EPs is their heterogeneity in size, density, surface markers, contents, and cell type of origin. The NIH Common Fund ExRNA Communication Consortium (ERCC) was established to examine the role of exRNA and associated carriers. ERCC aims to develop novel tools and technologies to isolate different EP classes and study their RNA cargo (Ainsztein *et al.* 2015, Das *et al.* 2019, Mateescu *et al.* 2022, https://doi.org/10.3402/jev.v4.27493, https://doi.org/10.1016/j.cell.2019.03.023, https://doi.org/10.1016/j.isci.2022.104653). While the EPs are orders of magnitude smaller (typically <150 nm) than a mammalian cell (typically > 10 µ), the EPs, like the cells from which they originate, carry surface markers recognizable by antibodies. Flow cytometry technologies have therefore been successfully repurposed to study EPs. Informally referred to as “nanoflow,” the repurposed flow cytometry experiments provide a wealth of data on distinct classes of EPs in complex mixtures and enable fast sorting of millions of EPs for downstream profiling of their cargo and other experimentation. However, nanoflow experiments are highly sensitive to experimental conditions because of EPs’ smaller size and immense diversity. Consequently, reproducing published results, validating or refuting previous findings, collaborating, and performing meta-analyses on published data becomes difficult. The MIFlowCyt-EV (Welsh *et al.* 2020a) framework helps address this problem by defining experimental metadata standards. The framework combines the existing Minimum Information for Studies of EVs (MISEV) guidelines (Théry *et al.* 2017) (https://doi.org/10.1080/20013078.2018.1535750) and Minimum Information about a flow cytometry experiment (MIFlowCyt) standard (Lee *et al.* 2008) (https://doi.org/10.1002/cyto.a.20623) for reporting metadata about experiment design, specimens, instrument configuration, and analysis parameters. However, the impact of the MIFlowCyt-EV framework has so far been limited by the lack of implementation, which we aim to address by developing the NanoFlow Repository.

## 2 Features

**Figure 1.**
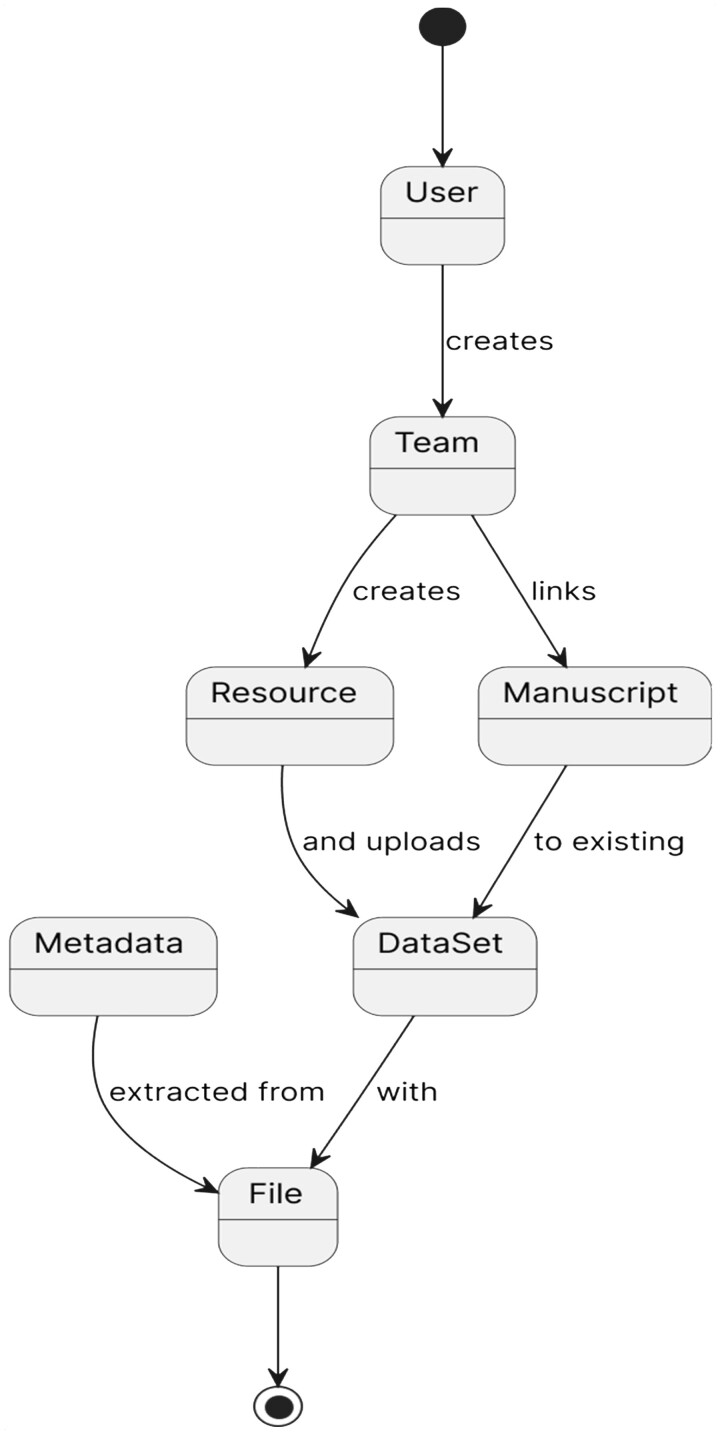
The data submission steps. Scientists can easily share and publish their nanoparticle datasets in simple steps. The data submitter, the user, creates a team. Acting on behalf of the team, the user made a resource that can have multiple datasets uploaded to it. A dataset comprises one or many FCS files generated from the same flow cytometer. File metadata is extracted, validated, transformed, and saved to the NanoFlow database.

The NanoFlow Repository utilizes standards and local data processing software tools. It also allows for eventual integration with the exRNA Atlas (Murillo *et al.* 2019) (https://doi.org/10.1016/j.cell.2019.02.018) into an atlas of EPs and their cargo. For example, single EP flow cytometry (EP-FC) data calibrated with FCM_PASS_ (Welsh *et al.* 2020) (https://doi.org/10.1002/cyto.a.23782) creates encrypted *.exRNA* files recognized by the repository to ensure data integrity. The NanoFlow Repository organizes uploaded FCS (Spidlen *et al.* 2010) (https://doi.org/10.1002/cyto.a.20825) files from the same flow cytometer into a dataset owned by a user-selected or user-created team and a MIFlowCyt-EV report. The Repository provides a MIFlowCyt-EV template in excel format as shown here https://genboree.org/nano-api/File/id/1415083238/data. Additionally, files from the calibration tools FCM_PASS_ and MPA_PASS_ (Welsh *et al.* 2022) (https://doi.org/10.1016/j.crmeth.2021.100136) are fully supported to compare data generated by different flow cytometers and resistive pulsar sensing technologies. Once all necessary data have been uploaded and validated, the team can publish their datasets to allow public access and download. Scientists can easily share and publish their EP-FC datasets by following the steps outlined in Fig. 1. The data submitter creates a new or selects an existing team to own the submitted data. Acting on behalf of the team, the user can organize datasets under a resource set and, optionally, a manuscript. With the resource set and optional manuscript, the user completes one or more dataset metadata forms and uploads FCS files, a MIFlowCyt-EV report, and, optionally, data analysis results.

## 3 Data model

**Figure 2.**
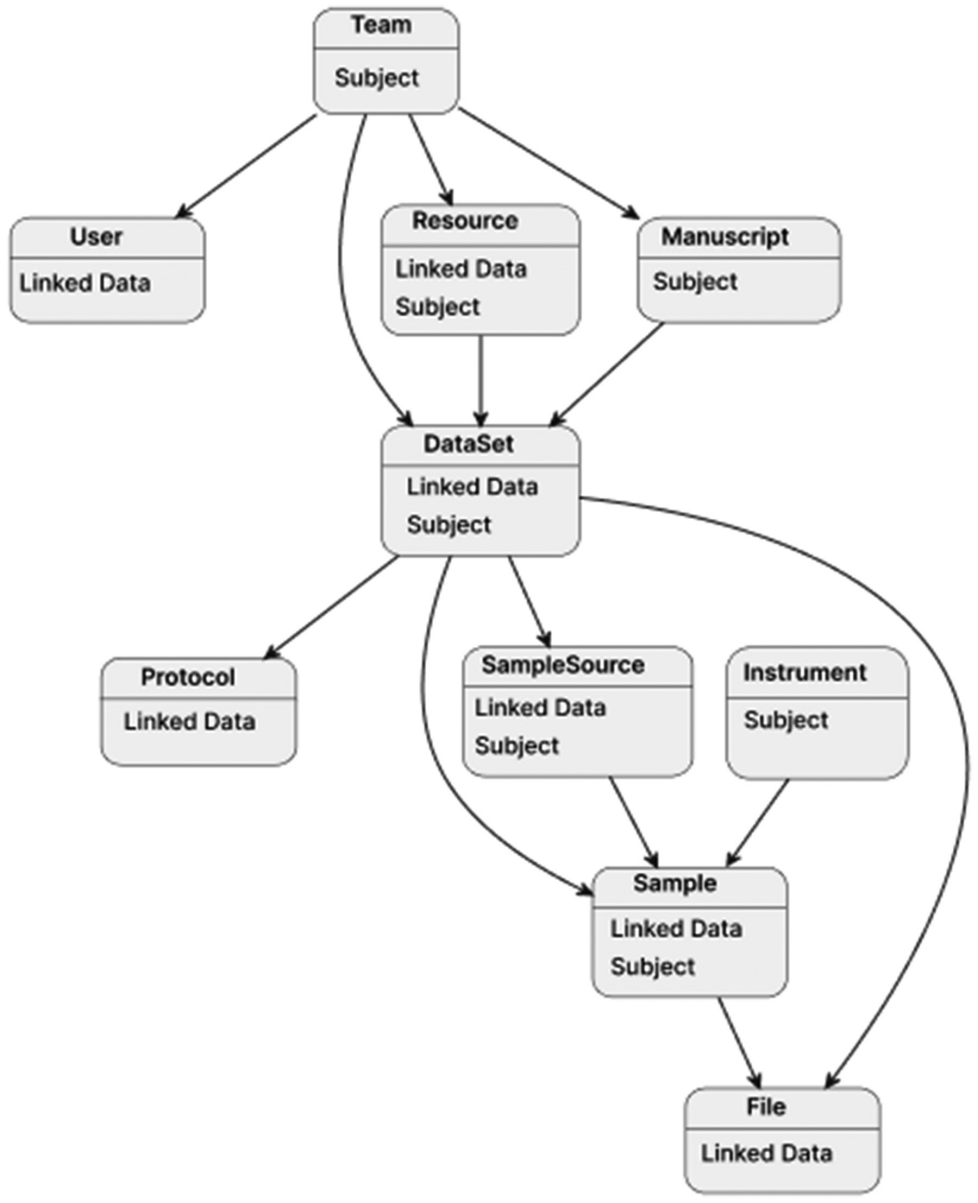
The graph entity data model. Each rounded rectangle represents a type of entity, e.g. Team, DataSet, or Sample; each entity is assigned the following roles: subject and linked data. Arrows represent edges that link entities to one another. The model is based on LDH’s subject and linked data entity roles to model data sources. Subject entities are related to linked data entities. For example, an entity of the type *Team* is a subject entity with user-linked data entities (members). Whereas an entity of the type *DataSet* is both a subject and linked data entity such that it is a linked data entity to a *Team* entity (the owners of the dataset) but a subject entity to a *SampleSource* entity.

The NanoFlow Repository models metadata as a graph of entity types, e.g. Team, DataSet, Sample; each entity is assigned both or one of the following roles: subject and linked data. Subject entities are related to linked data entities and edges are created from subject entities to linked data entities to establish entity relations. For example, an entity of type *Team* is a subject entity with user-linked data entities (members). Whereas an entity of type *DataSet* is both a subject and linked data entity such that it is a linked data entity to a *Team* entity (the owners of the dataset) but a subject entity to a *SampleSource* entity (Fig. 2).

## 4 Discussion

We have developed The NanoFlow Repository to provide the first implementation of the MIFlowCyt-EV framework. It enables sharing of EP-FC data and standards-compliant metadata about experimental design, samples, instrument configuration, and analysis parameters. The Repository will improve the rigor and reproducibility of EP research by enabling researchers to access nanoflow metadata and facilitate efforts to reproduce published results and validate previous findings. It will also catalyze collaboration and data reuse for meta-analyses. The Repository will allow EP researchers to develop Data Management and Sharing Plans per the recently articulated NIH Policy for Data Management and Sharing (Tabak 2020) (https://grants.nih.gov/grants/guide/notice-files/NOT-OD-21-013.html).

**Figure 3.**
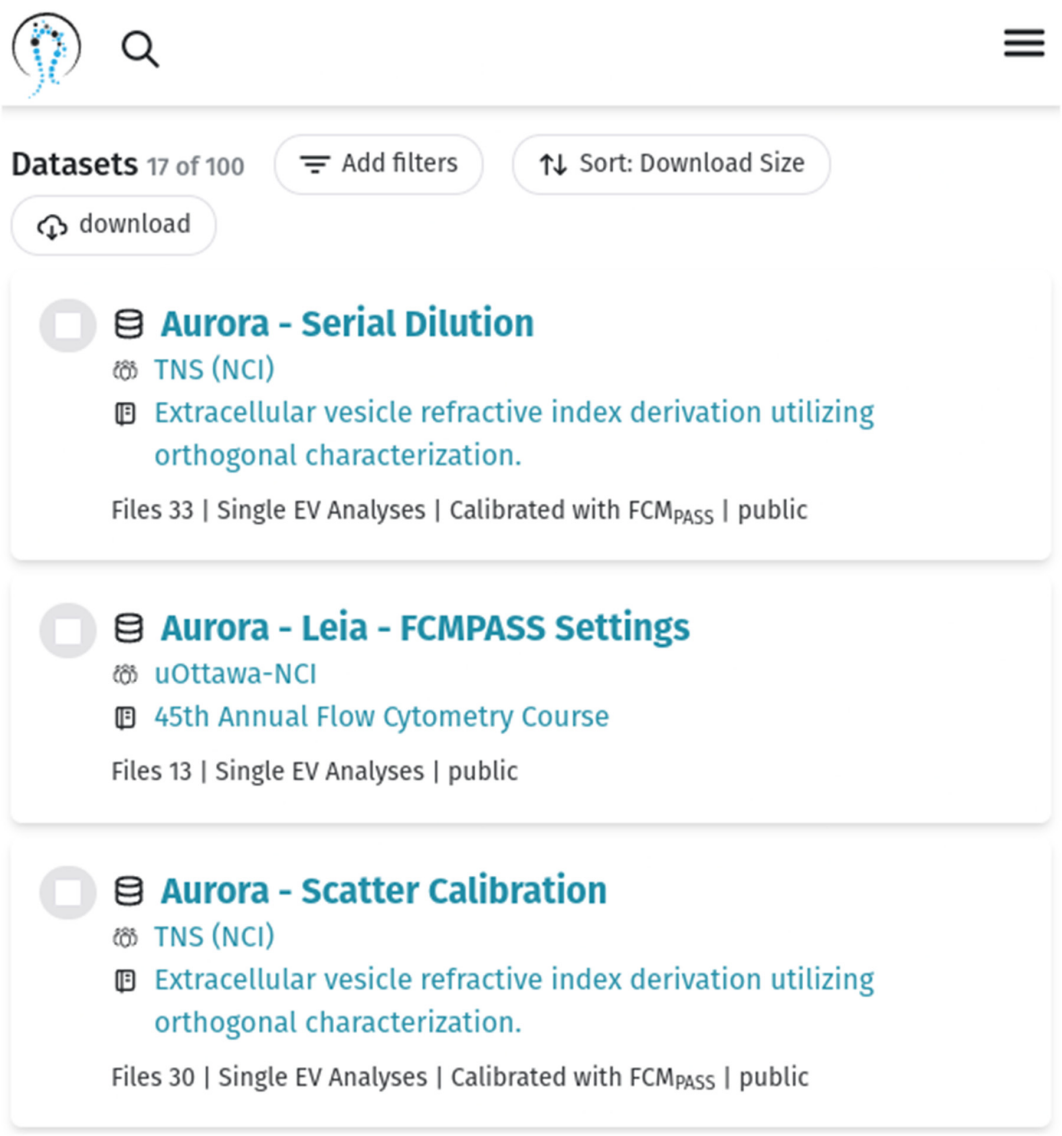
User interface listing publicly available NanoFlow datasets.

Currently, the NanoFlow Repository has 61 public datasets with 1723 FCS files created by various flow cytometers (Table 1). Public datasets can be searched, sorted, and filtered at https://genboree.org/nano-ui/ld/datasets?pgSize=40&pg=1 (Fig. 3). Furthermore, to support flow cytometer benchmarking, one can filter datasets by the type of flow cytometer used to generate the data. For example, the following link displays all datasets that contain FCS files generated from the Aurora flow cytometer: https://genboree.org/nano-ui/ld/datasets?filter=Sample.entContent.fcs.text.%24CYT%3AAurora&pgSize=50&pg=1.

**Table 1.** Usage and public content summary.

Publicly accessible content	Total/names
Datasets	61
FCS files	1723
Files (FCS, exRNA, xlsx, JSON)	2196
Teams	5
Manuscripts	4
Instruments	Aurora, CytoFLEX S, CytoFLEX LX, LSR Fortessa

Additionally, from the datasets listing page, one can follow a dataset’s link to examine the MIFlowCyt and MIFlowCyt-EV reports, samples, and download files. For example, the following link displays a dataset’s MIFlowCyt-EV and MIFlowCyt report, samples, and related data files: https://genboree.org/nano-ui/dataset/1313147970.

The scientific community may sign up for an account at https://genboree.org/nano-ui/auth/login. We invite the scientific community to explore and download data within the NanoFlow Repository and share EP-FC data by creating a team, uploading EP-FC data files, and linking a publication to submitted data.

## 5 Conclusion

NanoFlow Repository catalyzes research and collaboration into EPs by sharing nanoflow cytometry data and standards-compliant metadata about experiment design, specimens, instrument configuration, and analysis parameters, encouraging data reuse for meta-analyses and represents the first implementation of the MIFlowCyt-EV framework. Furthermore, by enabling researchers to reproduce published results and to validate or refute previous findings, the Repository will improve the rigor and reproducibility of EP research.

The NanoFlow Repository is one of many contributions toward the overarching Minimal Information for Biological and Biomedical Investigations effort to promote coherent minimum reporting guidelines for biological and biomedical investigations (Taylor *et al.* 2008) (https://doi.org/10.1038/nbt.1411). The significance of these efforts is widely recognized. Moreover, it is central to the success of the ongoing efforts by funding agencies to increase the impact of funded research projects through data sharing. Specifically, we anticipate that the Repository may enable EP researchers to develop Data Management and Sharing Plans per the recently articulated NIH Policy for Data Management and Sharing (Tabak 2020).

## Data Availability

All data is available here: https://genboree.org/nano-ui/ld/datasets.
